# Supplementary material for: Prevalence and determinants of precancerous cervical lesions among women screened for cervical cancer in Africa: A systematic review and meta-analysis
Source: PLoS One. 2025 Dec 10;20(12):e0338484. doi: 10.1371/journal.pone.0338484 (PMC12694816; doi:10.1371/journal.pone.0338484)
Supplement: S2 File — (DOCX) [file pone.0338484.s002.docx]

**Searching strategies**

**Google Scholar:**

"Prevalence" OR "magnitude" AND “precancerous AND cervical lesions” OR "premalignant cervical lesions" OR "pre-cancerous" AND "cervical lesion" OR "cervical intraepithelial neoplasia (CIN)” AND "Africa"

**PubMed**: (((((((Prevalence) OR (magnitude)) AND (precancerous)) AND (cervical lesions)) OR (pre-cancerous)) AND (cervical lesion)) OR (cervical intraepithelial neoplasia (CIN))) AND (Africa))))))

**Hinari**: (((((((Prevalence) OR (magnitude)) AND (precancerous)) AND (cervical lesions)) OR (pre-cancerous)) AND (cervical lesion)) OR (cervical intraepithelial neoplasia (CIN))) AND (Africa))))))

**Science Direct:** (((((((Prevalence) OR (magnitude)) AND (precancerous)) AND (cervical lesions)) OR (pre-cancerous)) AND (cervical lesion)) OR (cervical intraepithelial neoplasia (CIN))) AND (Africa))))))
