# Supplementary material for: Prevalence and determinants of precancerous cervical lesions among women screened for cervical cancer in Africa: A systematic review and meta-analysis
Source: PLoS One. 2025 Dec 10;20(12):e0338484. doi: 10.1371/journal.pone.0338484 (PMC12694816; doi:10.1371/journal.pone.0338484)
Supplement: S4 File — (DOCX) [file pone.0338484.s004.docx]

Table 4: A leave-one-out sensitivity analysis for precancerous cervical lesions among women who screened in Africa.

| Omitted study | Effect size | [95% confidence  interval] | | P-value |
| --- | --- | --- | --- | --- |
| Abera et al, 2021 | 17.138 | 15.540 | 18.735 | 0.000 |
| Adam et al, 2022 | 16.835 | 15.247 | 18.424 | 0.000 |
| Adam et al, 2022 | 17.172 | 15.579 | 18.765 | 0.000 |
| Ago et al., 2016 | 17.188 | 15.595 | 18.782 | 0.000 |
| Ali et al, 2019 | 17.095 | 15.501 | 18.689 | 0.000 |
| Ambounda‑Ledaga .., 2024 | 16.968 | 15.377 | 18.559 | 0.000 |
| Awolude et al., 2021 | 17.126 | 15.530 | 18.721 | 0.000 |
| Bateman et al, 2015 | 16.611 | 15.041 | 18.180 | 0.000 |
| Belayneh et al, 2019 | 17.125 | 15.531 | 18.719 | 0.000 |
| CHIBVONGODZE et al, 2017 | 17.088 | 15.498 | 18.678 | 0.000 |
| Chris-Ozoko et al., 2020 | 17.161 | 15.557 | 18.765 | 0.000 |
| Christensen et al, 2023 | 17.161 | 15.548 | 18.774 | 0.000 |
| Daniel et al., 2019 | 17.103 | 15.509 | 18.698 | 0.000 |
| Darré et al, 2024 | 17.110 | 15.517 | 18.704 | 0.000 |
| Deksissa et al, 2015 | 17.097 | 15.503 | 18.691 | 0.000 |
| Derbie et al, 2022 | 17.039 | 15.447 | 18.632 | 0.000 |
| Desire et al, 2016 | 16.880 | 15.291 | 18.469 | 0.000 |
| Dibisa et al, 2022 | 16.963 | 15.373 | 18.554 | 0.000 |
| Diop et al, 2022 | 17.169 | 15.574 | 18.764 | 0.000 |
| Doh et al, 2021 | 16.969 | 15.379 | 18.560 | 0.000 |
| Donkhon et al., 2019 | 17.187 | 15.592 | 18.782 | 0.000 |
| Effah et al., 2024 | 17.138 | 15.544 | 18.732 | 0.000 |
| Effah et al., 2024 | 17.106 | 15.513 | 18.698 | 0.000 |
| Eljabuet al, 2021 | 17.184 | 15.589 | 18.779 | 0.000 |
| Eseoghene, et al 2021 | 17.062 | 15.468 | 18.655 | 0.000 |
| Essmat et al, 2021 | 17.059 | 15.465 | 18.654 | 0.000 |
| Fentie et al, 2020 | 17.125 | 15.528 | 18.721 | 0.000 |
| Getinet et al, 2021 | 17.075 | 15.481 | 18.668 | 0.000 |
| Getinet et al, 2024 | 17.087 | 15.493 | 18.681 | 0.000 |
| GETINET et al, 2024 | 17.088 | 15.494 | 18.681 | 0.000 |
| Gnatou et al, 2024 | 17.186 | 15.590 | 18.781 | 0.000 |
| Hailemariam et al, 2017 | 17.066 | 15.468 | 18.663 | 0.000 |
| Hailemariam et al, 2020 | 17.135 | 15.540 | 18.730 | 0.000 |
| Hayumbu et al, 2021 | 17.040 | 15.448 | 18.633 | 0.000 |
| Hoffman et al, 2016 | 16.932 | 15.342 | 18.522 | 0.000 |
| Ibrahima et al., 2023 | 17.136 | 15.532 | 18.740 | 0.000 |
| Inuwa et al., 2016 | 16.780 | 15.198 | 18.363 | 0.000 |
| Inuwa et al., 2016 | 16.925 | 15.336 | 18.514 | 0.000 |
| Irabor et al., 2018 | 17.059 | 15.465 | 18.653 | 0.000 |
| Jolly et al, 2017 | 17.006 | 15.414 | 18.598 | 0.000 |
| Jolly et al, 2017 | 17.165 | 15.571 | 18.760 | 0.000 |
| Kagoné et al., 2022 | 17.074 | 15.480 | 18.669 | 0.000 |
| Kamdem et al, 2022 | 16.797 | 15.210 | 18.384 | 0.000 |
| Karuri et al, 2015 | 16.915 | 15.325 | 18.504 | 0.000 |
| Kaseka et al, 2022 | 16.990 | 15.398 | 18.581 | 0.000 |
| Kassa LS et al, 2019 | 17.029 | 15.436 | 18.622 | 0.000 |
| Katz et al., 2016 | 16.533 | 14.969 | 18.096 | 0.000 |
| Kirabira et al, 2024 | 17.007 | 15.415 | 18.598 | 0.000 |
| Kirabira et al, 2024 | 17.104 | 15.510 | 18.697 | 0.000 |
| Kiros et al, 2021 | 17.132 | 15.537 | 18.727 | 0.000 |
| Kiros et al, 2021 | 17.133 | 15.540 | 18.727 | 0.000 |
| Kurtay et al, 2022 | 17.104 | 15.509 | 18.699 | 0.000 |
| Lawal et al., 2017 | 16.756 | 15.170 | 18.341 | 0.000 |
| Lawal et al., 2017 | 17.097 | 15.504 | 18.690 | 0.000 |
| Ledaga et al, 2022 | 17.104 | 15.511 | 18.697 | 0.000 |
| Lemma et al, 2024 | 17.068 | 15.475 | 18.661 | 0.000 |
| Lemu et al, 2021 | 17.043 | 15.450 | 18.636 | 0.000 |
| Macharia et al, 2017 | 17.017 | 15.426 | 18.608 | 0.000 |
| Magaji et al, 2024 | 16.993 | 15.402 | 18.584 | 0.000 |
| Makuza et al, 2015 | 17.203 | 15.615 | 18.790 | 0.000 |
| Mariko et al, 2021 | 17.187 | 15.585 | 18.789 | 0.000 |
| Mayeri et al., 2024 | 17.059 | 15.466 | 18.651 | 0.000 |
| Mekuria et al, 2021 | 16.999 | 15.407 | 18.590 | 0.000 |
| Merera et al, 2021 | 17.071 | 15.477 | 18.664 | 0.000 |
| Misgina et al, 2016 | 17.155 | 15.560 | 18.749 | 0.000 |
| Mremi et al, 2022 | 17.054 | 15.459 | 18.649 | 0.000 |
| Muia et al., 2021 | 17.146 | 15.551 | 18.741 | 0.000 |
| Mukanyangezi et al, 2018 | 16.992 | 15.400 | 18.583 | 0.000 |
| Mukanyangezi et al, 2018 | 17.126 | 15.531 | 18.721 | 0.000 |
| Mulugeta Y., 2022 | 17.063 | 15.470 | 18.657 | 0.000 |
| Mulugeta Y., 2022 | 17.129 | 15.534 | 18.723 | 0.000 |
| Musa et al., 2020 | 17.189 | 15.592 | 18.787 | 0.000 |
| MUTUKU et al, 2018 | 17.146 | 15.551 | 18.741 | 0.000 |
| Ngwibete et al., 2024 | 17.114 | 15.520 | 18.707 | 0.000 |
| Njagi et al, 2021 | 16.833 | 15.248 | 18.419 | 0.000 |
| Njagi et al, 2021 | 17.042 | 15.451 | 18.634 | 0.000 |
| Nkfusai et al, 2017 | 17.181 | 15.588 | 18.774 | 0.000 |
| Ntuliet et al, 2020 | 16.400 | 15.318 | 17.481 | 0.000 |
| Ntuliet et al, 2020 | 17.233 | 15.446 | 19.020 | 0.000 |
| Nzang et al., 2024 | 17.091 | 15.498 | 18.685 | 0.000 |
| Oduor et al., 2016 | 16.725 | 15.148 | 18.301 | 0.000 |
| Okorie et al., 2017 | 17.117 | 15.524 | 18.711 | 0.000 |
| OKUNADE et al., 2023 | 17.158 | 15.562 | 18.754 | 0.000 |
| Okunowo et al., 2023 | 17.125 | 15.531 | 18.719 | 0.000 |
| Okwi et al, 2017 | 17.117 | 15.519 | 18.715 | 0.000 |
| Omeke et al., 2022 | 17.077 | 15.483 | 18.670 | 0.000 |
| Omoragbon et al., 2017 | 16.996 | 15.406 | 18.587 | 0.000 |
| Omoragbon et al., 2017 | 17.059 | 15.468 | 18.651 | 0.000 |
| Omoyeni et al, 2022 | 16.792 | 15.207 | 18.377 | 0.000 |
| Oringo J., 2020 | 17.164 | 15.570 | 18.758 | 0.000 |
| Oumar et al, 2022 | 17.230 | 15.450 | 19.009 | 0.000 |
| Paluku et al., 2019 | 17.151 | 15.555 | 18.748 | 0.000 |
| Rantshabeng et al., 2024 | 17.090 | 15.496 | 18.683 | 0.000 |
| Siad et al, 2023 | 17.072 | 15.477 | 18.667 | 0.000 |
| Simo et al, 2021 | 17.059 | 15.466 | 18.651 | 0.000 |
| Simo et al, 2021 | 17.073 | 15.480 | 18.666 | 0.000 |
| Ssedyabane et al, 2024 | 17.158 | 15.563 | 18.752 | 0.000 |
| Stroetmann et al, 2024 | 17.193 | 15.534 | 18.852 | 0.000 |
| Teame et al, 2018 | 17.098 | 15.504 | 18.692 | 0.000 |
| Temesgen et al, 2021 | 17.095 | 15.501 | 18.689 | 0.000 |
| Temesgen et al, 2019 | 17.155 | 15.560 | 18.750 | 0.000 |
| Temesgen et al, 2020 | 17.085 | 15.492 | 18.678 | 0.000 |
| Tenkir et al, 2023 | 17.087 | 15.493 | 18.681 | 0.000 |
| Tirkaso et al, 2024 | 17.063 | 15.465 | 18.661 | 0.000 |
| Ugboaja et al., 2016 | 16.973 | 15.382 | 18.564 | 0.000 |
| Umemmuo MU et al 2019 | 17.175 | 15.556 | 18.793 | 0.000 |
| Vieira et al., 2024 | 17.072 | 15.458 | 18.686 | 0.000 |
| Wabo et al, 2022 | 17.106 | 15.510 | 18.703 | 0.000 |
| Wakwoya et al, 2020 | 16.985 | 15.396 | 18.575 | 0.000 |
| Worku et al, 2024 | 16.986 | 15.396 | 18.577 | 0.000 |
| WoromogoIet al, 2021 | 17.011 | 15.419 | 18.603 | 0.000 |
| Zelalem et al, 2022 | 17.148 | 15.554 | 18.742 | 0.000 |
| Overall estimate | 17.058 | 15.472 | 18.643 | 0.000 |
