# Supplementary material for: Prevalence and determinants of precancerous cervical lesions among women screened for cervical cancer in Africa: A systematic review and meta-analysis
Source: PLoS One. 2025 Dec 10;20(12):e0338484. doi: 10.1371/journal.pone.0338484 (PMC12694816; doi:10.1371/journal.pone.0338484)
Supplement: Attachment — Submitted filename: Response to Reviewers.docx [file pone.0338484.s006.docx]

***August 08, 2025***

**Prevalence and determinants of precancerous cervical lesions among women screened for cervical cancer in Africa: a systematic review and meta-analysis**

**Manuscript number: PONE-D-25-16143**

Dear **Kazunori Nagasaka (Professor, Academic Editor),** We are delighted to receive your comments for our submission. We are also grateful for the honored reviewers; their scientific comments, expertise, and guidance have been vital in enhancing the overall quality of our paper. We have diligently addressed the raised comments and suggestions given by the reviewer. The changes have been fully included in the revised manuscript. Finally, we kindly invite you to take a look at a point-by-point response below:

**Point-by-point response to Reviewers’ comments**

**Reviewer #1**: This manuscript addresses an important public health concern in Africa. However, several aspects need improvement to meet the standard of a high-quality systematic review and meta-analysis, especially for publication in PLOS ONE. Below are my comments and suggestions:

**Authors’ response: Dear Reviewer,** we would like to express our sincere gratitude for your valuable time and constructive feedback on our manuscript. We greatly appreciate your insightful comments and for sharing your expertise to improve our work. In response to the reviewer’s thoughtful comments and suggestions, we have carefully and thoroughly addressed each of the points raised. As a result, we carried out a substantial revision throughout the manuscript, specifically, and we have addressed other concerns raised by the reviewer.

**Reviewer’s comment: Abstract:**

1. Lines 31-34: The sentence is redundant and grammatically incorrect. Please revise it for clarity and conciseness.

**Authors’ response: Thank you!** We have removed the redundant sentence and checked for grammar.

**Reviewer’s comment:** 2. Google Scholar and ScienceDirect are not considered formal scientific databases. They are scientific search engines. Please revise accordingly and clarify the actual databases used.

**Authors’ response: Thank you for the comment.** We have revised and clarify accordingly.

**Reviewer’s comment:** 3. Please include the statistical methods used to estimate the pooled prevalence in the abstract.

**Authors’ response: Thank you.** We have mentioned the statistical methods used to estimate the pooled prevalence in the abstract section. i.e., **a random-effects model using the Der Simonian Laird method.**

**Reviewer’s comment:** 4. The conclusion in the abstract is too lengthy. Kindly, condense it to focus on the key findings and implications.

**Authors’ response:** We appreciate your insightful comment. We have revised the conclusion part of the abstract to make it concise.

***Introduction***

**Reviewer’s comment:**  1. Since many countries already have cervical cancer care cascade data, please justify the rationale for conducting this study, especially with respect to screening data. What specific gap does this study aim to address?

**Authors’ response: Thank you for this valuable comment.** Even though many countries have cervical cancer care cascade data, there remains a critical gap in a comprehensive or consolidated evidence regarding precancerous cervical lesions among women undergoing screening in African context. Most existing datasets focus on incidence and mortality of invasive cervical cancer, HPV vaccination coverage, or general screening uptake, but there is scarcity of evidence on the magnitude of precancerous lesions. This meta-analysis aimed to fill that gap by synthesizing fragmented studies to better understand the burden at the early, detectable stage of the disease. Generating such evidence is crucial for evaluating the effectiveness of screening programs and taking early intervention strategies, especially as precancerous lesions represent a key target for timely treatment and prevention of cervical cancer.

**Reviewer’s comment:** 2. The objectives section mentions identifying factors associated with precancerous cervical lesions. Please clarify why identifying these factors is important. Are there any previous systematic reviews on this topic? If so, how does this study build on or differ from them?

**Authors’ response:** **Dear Reviewer, thank you so much for raising this important point.** This review also identified and analyzed associated factors of precancerous cervical lesions, which is critical primarily to tackle modifiable predictors of PCL. Existing systematic reviews on precancerous cervical lesions are limited in number and predominantly conducted in Ethiopia context. In contrast, the current review expands both the scope and geographical context by incorporating a broader range of studies and sample size across Africa. This enhances the generalizability and robustness of the findings, providing more comprehensive evidence regarding the burden and associated factors of precancerous cervical lesions in Africa.

**Reviewer’s comment:** Please clearly justify the focus on African countries. What specific contextual or epidemiological reasons necessitate this geographical limitation?

**Authors’ response:** **Thank you.** We have made substantial revision the main triggers for conducting this review. Please, take a look in the revised manuscript.

The focus on African countries is driven by both epidemiological and contextual disparities in cervical cancer prevention and treatment. Africa accounts a high burden of cervical cancer, with some of the highest global incidence and mortality rates. This could be associated with low screening coverage, limited access to early detection and treatment of PCL, which result in delayed diagnosis and a higher likelihood of progression to invasive cancer. By focusing on African countries, this review aimed to highlight targeted interventions, optimize screening strategies, and support the implementation of WHO's cervical cancer elimination initiative across African countries.

**Reviewer’s comment:** The research question should be more clearly integrated with the stated objective(s) of the study.

**Authors’ response:** Thank you for your insightful comment. Accordingly, we have modified the title and changes has been done throughout the manuscript. Thus, the research topic is consistent with the research questions.

**Reviewer’s comment:** **Methods:**

1. If the study used the Co Co Pop framework (Condition, Context, Population) to define eligibility criteria, please state this explicitly. Each component should be clearly defined. For example, does the population include all women regardless of HIV status, age, or other criteria?
**Authors’ response:** Thank you very much for this scientific comment. Accordingly, we have updated the Co Co Pop framework in the revised manuscript clearly.

**Reviewer’s comment:** 2. Please cite relevant tools/software appropriately. For instance, if EndNote 20 and STATA 17 was used, a proper citation or acknowledgment should be included.

**Authors’ response:** **Thank you!** We have acknowledged Citation manager (EndNote) and Statistical analysis software (STATA 17) in the revised manuscript.

**Reviewer’s comment:** 3. Specify the statistical methods used to calculate the adjusted odds

ratios (AORs) for associated factors.

**Authors’ response:** Thank you for your valuable comments. For the meta-analysis of factors associated

with PCL, we extracted adjusted odds ratios (AORs) from studies that reported results from multivariable

logistic regression analyses, including only factors reported in ≥2 studies. The raw AORs were converted to

log odds ratios in Excel Spreadsheet. We then exported the data to Stata version 17 and performed a

random-effects meta-analysis using the Der Simonian and Laird method to account for between-study

heterogeneity. The pooled AORs and their 95% confidence intervals were reported.

**Reviewer’s comment: Results:**

1. Table 1 is not reader-friendly. The information is too dense. Please consider breaking it into clusters or themes for better clarity.

**Authors’ response: Thank you for the comment.** We made some amendments on Table 1,

including arranging studies in alphabetical order and citation.

**Reviewer’s comment:** 2. I believe the study design and mean age data are important to presented in Table 1.

**Authors’ response: Thank you for your suggestion.** We have presented the study design and mean or median age in Table 1. We kindly invite to look at the revised manuscript.

**Reviewer’s comment**: 3. Figure 1 (PRISMA flow diagram) needs improvement in terms of aesthetics and readability. Please revise using standard PRISMA templates.

**Authors’ response:** We made amendment on the PRISMA flow diagram.

**Reviewer’s comment:** 4. Lines 223-224: Please cite included studies “In this 224 systematic review and meta-analysis, 112 eligible articles”

**Authors’ response: Thank you.** We have cited all references in the result section and in the summary table 1.

**Reviewer’s comment: 5. Table 4 would be more appropriate as a supplementary file to enhance the flow of the main manuscript.**

**Authors’ response:** We have uploaded **“Table 4”** as Supplementary file.

**Reviewer’s comment:** 6. Lines 266-267 and Table 5: One of the variables is reported with a wide confidence interval (AOR = 4.07, 95% CI: 1.74–9.53). This suggests high uncertainty or possible heterogeneity in the pooled effect estimate. In fact, many variables in the table show similarly wide intervals, suggesting that the pooled effect estimates may be unstable or based on small sample sizes or a limited number of studies. Please address this issue in the Discussion section by (1) Acknowledging the presence of wide confidence intervals across several variables. (2) Discussing potential reasons for this (e.g., variation in study design, measurement, sample size, or context). (3) Considering sensitivity or subgroup analyses to explore and explain the heterogeneity.

**Authors’ response:** we sincerely appreciate your constructive feedback and valuable suggestions aimed at improving the quality of our manuscript. Accordingly, we acknowledged and discussed the presence of slightly wide confidence intervals (CIs) for some factors, indicating uncertainty in the pooled estimates. To address potential heterogeneity across studies, we performed sensitivity and subgroup analyses and discussed all plausible sources contributing to the observed variation.

**Reviewer’s comment: *Discussion:***

1. The discussion would benefit from further elaboration on the implications of the findings, especially regarding regional and national cervical cancer screening policies in Africa. How can policymakers or health systems use this evidence?

**Authors’ response: Thank you very much for this insightful suggestion.** As per your recommendation, (1) we have elaborated on the implications of the key findings, and (2) we have clearly presented the overall implications of the review in the revised manuscript.

**Reviewer’s comment:** 2. Given that PubMed appears to be the primary database searched, there is a risk of publication bias. This limitation should be explicitly discussed in the manuscript, including its potential influence on the findings.

**Authors’ response: Thank you once again.** We have stated the use of PubMed as a main database for literature search. PubMed is a widely recognized biomedical database for accessing indexed published articles. To ensure comprehensive searching and bridge potential gap for using PubMed, we augmented our search by systematically exploring Google Scholar to identify both published and unpublished studies, and we also used ScienceDirect to retrieve relevant articles from Elsevier-indexed journals.

**Reviewer’s comment: Reference**

There appears to be a discrepancy in the reporting of included studies and referenced articles. You mentioned that 112 studies were included in the review, but only 72 studies are listed in the reference section.

**Authors’ response: Thank you in advanced for your scientific and insightful comment.** Thus, we have cited all the included references at the beginning of the result section. Additionally, all articles are acknowledged in the “Table 1”. We kindly invite to take a look at the revised manuscript.

**Thank you in advance!!**

**Reviewer #2: Thank you for the opportunity to read this manuscript.**

**Reviewer’s comment:** It is a very thorough and extensive approach to a very important topic, cervical cancer screening in LMI countries. As obvious from the included papers in the review and meta-analyses, the topic in complicated due to a number of different screening methods, varying study population size, HIV status over the huge continent.

**Authors’ response: Dear Reviewer,** we appreciate your general and specific comments for our manuscript, which serve as an input to improve the overall quality of our paper. **As the reviewer stated, we believe that we have conducted a systematic review in one of the public health topics of the continent. To address your comment, we have attempted to follow the following:**

1) **Comprehensive and systematic searching strategy to access the eligible articles**
2) **Set clear eligibility criteria and framework (CoCo Pop)**
3) **Operationalize “precancerous cervical lesions (PCL)”.** The current review included all studies that reported positive findings for PCL, regardless of women’s HIV status, or the cervical cancer screening methods employed.
4) **Appropriate data extraction and statistical analyses**
5) **Results described and findings discussed with their implications**

**Reviewer’s comment:** Unfortunately, I have issues concerning the manuscript. I think the introduction itself could be more condensed.

**Authors’ response: Thank you for this insightful comment.** We have accepted the reviewer’s suggestion regarding the Introduction section. Accordingly, we made substantial revisions to improve the flow and coherence of the problem of statement. We kindly invite you to see the manuscript with tracked changes and the revised manuscript.

**Reviewer’s comment:** I do not see any clear definition of precancerous lesion, i.e. does it include only HSIL lesion in cytology, does it include other cytological diagnosis, does it mean the presence of hrHPV alone.

**Authors’ response: Dear Reviewer, thank you for raising this vital point, which directly related to the outcome of interest in our systematic review.** Honestly speaking, we have attempted to define “precancerous lesions” in the second paragraph of the introduction **[Line 74-79].** Additionally, we made revision and cited with appropriate references. We have operationalized precancerous lesions based on different screening method approved by WHO, including Pap Smear, HPV DNA test, cytology or Visual inspections methods to diagnose PCL. Please, take a look on the revised manuscript. The presence of High-risk human papillomavirus (hrHPV) is not alone used to diagnose PCL or CIN, rather the woman should be positive by available screening methods.

**Reviewer’s comment:** And foremost, which new knowledge compared to the state of the art does this manuscript ad?

**Authors’ response: Finally, the authors sincerely appreciate the reviewer’s comment regarding the relevance of the review and what it adds.**

The following are the main significances of this review:

1. **This systematic review and meta-analysis determined the burden of precancerous cervical lesions or** cervical intraepithelial neoplasia (CIN) **in Africa. Precancerous cervical lesions or** CIN are precancerous conditions or precursors of invasive cervical cancer. The findings of this review indicate the need for implementing evidence-based interventions for preventing advanced stages of cervical cancer at national or regional levels. It highlights that the governmental and non-governmental bodies should collaborate to strengthen the health system, expanding HPV vaccination, capacity of cervical screening programs, timely diagnosis and treatment of PCL to prevent long-term consequences of cervical cancer.
2. In addition, this meta-analysis also identified and analyzed several modifiable factors that associated with PCL. This helps to take targeted and context-specific interventions to prevent PCL and eventually cervical cancer. For instance, expanding education (school-based, community education), counseling on safe sexual behavior and practices, unaddressed areas (rural dwellers), giving attention to marginalized and high-risk groups (HIV patients) etc.,
3. In this meta-analysis, the magnitude of PCL primarily based on results of facility-based studies in the continent. That means, the burden of PCL could be underestimated. Therefore, it is highly recommended to expand both facility-based and community based cervical cancer screening for early detection and treatment of PCL.

**In sum,** the findings can serve as a baseline evidence and offer valuable insights for clinicians, policymakers, researchers, and other stakeholders (NGOs, funders) to prioritize the prevention and treatment of precancerous cervical lesions (precursor of invasive cervical cancer).

**Thank you!!**
