# Supplementary material for: Prevalence and determinants of precancerous cervical lesions among women screened for cervical cancer in Africa: A systematic review and meta-analysis
Source: PLoS One. 2025 Dec 10;20(12):e0338484. doi: 10.1371/journal.pone.0338484 (PMC12694816; doi:10.1371/journal.pone.0338484)
Supplement: Attachment — Submitted filename: Response_to_Reviewers_auresp_2.docx [file pone.0338484.s007.docx]

***October 07, 2025***

**Prevalence and determinants of precancerous cervical lesions among women screened for cervical cancer in Africa: a systematic review and meta-analysis**

**Manuscript number: PONE-D-25-16143**

Dear **Kazunori Nagasaka (Professor, Academic Editor),** we are delighted to receive your comments for our submission. We are also grateful for the honored reviewers; their scientific comments, expertise, and guidance have been vital in enhancing the overall quality of our paper. We have diligently addressed the raised comments and suggestions given by the reviewer. The changes have been fully included in the revised manuscript. Finally, we kindly invite you to take a look at a point-by-point response below:

**Point-by-point response to Reviewers’ comments**

**Reviewer #1**: Thank you for your thoughtful revisions. We truly appreciate the improvements you made and are pleased with the updated version of the manuscript.

**Authors’ response:** Dear reviewer #1, we would like to express our gratitude for sharing your time and expertise to reviewing our paper. Your comments and suggestions were fundamental inputs to improve the quality of our manuscript.

**Reviewer #1**: As this meta-analysis involves many included studies, we kindly suggest carefully checking for any errors during the next stage, especially in citations and references, since some issues may arise during the galley proof stage. We believe that ensuring accuracy is the authors’ responsibility.

**Authors’ response:** Absolutely! Thank you for your insightful comment related to appropriate referencing. We make a double check on the citations and references.

**Thank you in advance!!**

**Reviewer #2**: I have with interest taken part of the revised manuscript.

The area of the paper is still, or even now more, important in the light of recent developments concerning the availability concerning antiviral therapy in SSA and its potential of an even more critical situation in cervical cancer morbidity and mortality.

However, some of my concerns still prevail.

Concerning the introduction, it would benefit from a more comprehensive and structured approach.
As a minor comment PCCL is in a few places used instead of PCL in the manuscript.

**Authors’ response:** You honored reviewer #2, thank for your time and constructive comments on our manuscript.

**Reviewer’s comment:** Concerning especially the meta-analyses, I have concerns concerning the

lack of diagnostic stringency (**ref**. 49) in at least one of the rather few original articles I have read

myself, and as far as I understand LSIL is included in the PCL outcome of other studies (**ref. 51**)

whilst only histological CIN2+ after VIA and/or HPV screening seems to be the included

outcome concerning ref 53, however the figures might need to be checked.

**Authors’ response: Thank you for the comments.** Minor topographical errors are corrected. We critically take a look the figures of **Ref. 49, 52 and 53** for its consistency and validity. Let’s explain what actually we did, we included studies that report the overall magnitude of PCL that ranges from mild (CIN 1 or LSIL) to more severe (CIN 2, CIN 3 or HSIL). For instance, in Ref. 49, the magnitude of overall PCL is

**Reviewer’s comment:** My point is not some minor possible error in the statistical material, but the problem doing meta-analyses on such a complex material with different screening methods (VIA, HPV, cytology) and different approaches using these alone or in combination, is it even valid in the medical perspective.

Thus, at least this must be further and in depth discussed in the limitations of the study.

**Authors’ response:** We appreciate the reviewer’s concern regarding the inclusion of studies with different diagnostic (screening) methods. A mount of evidence has shown the presence of high burden of advanced stage of cervical cancer is Africa, particularly in SSA. Precancerous cervical lesions or CIN are the premalignant cervical conditions that progress into advanced stages. Therefore, initially the authors intended and triggered to determine the overall magnitude of this precursor condition by combining pre-existing studies. The included studies utilized different screening techniques recommended by WHO with relatively varied degree of accuracy. We acknowledge this variation may result in substantial heterogeneity in the pooled estimate and limit the generalizability of the finding.

There are few similar studies that aggregate the magnitude of PCL diagnosed by different screening methods. For instance: **Precancerous cervical lesion in Ethiopia: systematic review and meta-analysis, DOI: https://doi.org/10.1186/s13643-021-01840-0**

With its limitations, the authors believe that this figure can serve as conclusive baseline evidence for healthcare professionals, policymakers, researchers and non-governmental organizations. This trigger for expanding both primary prevention (HPV vaccinations) and secondary prevention approaches; inclusive cervical cancer screening (facility-based and community-based screening) for timely diagnosis and treatment of PCL. In addition, some systematic review and meta-analysis studies followed similar approach. Finally, as per the reviewer’s suggestion, since we have conducted a secondary data analysis we acknowledge and discussed the limitations of this review.

**Thank you!**
